# Prediction of Lysine Ubiquitylation with Ensemble Classifier and Feature Selection

**DOI:** 10.3390/ijms12128347

**Published:** 2011-11-28

**Authors:** Xiaowei Zhao, Xiangtao Li, Zhiqiang Ma, Minghao Yin

**Affiliations:** 1 College of Life Science, Northeast Normal University, 5268 Renmin Street, Changchun 130024, China; E-Mail: zhaoxw303@nenu.edu.cn; 2 College of Computer Science, Northeast Normal University, 2555 Jingyue Street, Changchun 130117, China; E-Mail: lixt314@nenu.edu.cn

**Keywords:** ubiquitylation, ensemble classifier, support vector machine, lysine ubiquitylation sites

## Abstract

Ubiquitylation is an important process of post-translational modification. Correct identification of protein lysine ubiquitylation sites is of fundamental importance to understand the molecular mechanism of lysine ubiquitylation in biological systems. This paper develops a novel computational method to effectively identify the lysine ubiquitylation sites based on the ensemble approach. In the proposed method, 468 ubiquitylation sites from 323 proteins retrieved from the Swiss-Prot database were encoded into feature vectors by using four kinds of protein sequences information. An effective feature selection method was then applied to extract informative feature subsets. After different feature subsets were obtained by setting different starting points in the search procedure, they were used to train multiple random forests classifiers and then aggregated into a consensus classifier by majority voting. Evaluated by jackknife tests and independent tests respectively, the accuracy of the proposed predictor reached 76.82% for the training dataset and 79.16% for the test dataset, indicating that this predictor is a useful tool to predict lysine ubiquitylation sites. Furthermore, site-specific feature analysis was performed and it was shown that ubiquitylation is intimately correlated with the features of its surrounding sites in addition to features derived from the lysine site itself. The feature selection method is available upon request.

## 1. Introduction

Ubiquitylation is a universal and important post-translational modification where ubiquitin is linked to some lysine residues of target proteins [1–3], and forms an isopeptide bond between the ɛ-amino groups of lysine residues of a substrate protein and the *C*-terminal double-glycine carboxy groups of ubiquitin protein [4,5]. Note that only the last glycine of ubiquitin is linked to substrate lysine residues in this process. There are mainly three kinds of enzymes participating in this highly collaborative process, including ubiquitin-activating enzymes, ubiquitin-conjugating enzymes, and ubiquitin ligases [6,7]. During the past decade, the function of ubiquitylation has been extended far beyond its role in just directing protein degradation [1,2], for example to the control of signal transduction, the regulation of DNA repair and transcription, and the implication of endocytosis and sorting [8].

Since identification of protein lysine ubiquitylation sites is of fundamental importance to understand the molecular mechanism of lysine ubiquitylation in biological systems, many post-genome era researchers have focused on this field [9–11]. Meanwhile, some high-throughput experimental technologies have been developed to analyze and model the lysine ubiquitylation process at a genomic scale, such as proteolytic digestion, three steps affinity purification, and analysis using mass spectrometry [12]. However, these conventional experiment approaches are labor-intensive and time-consuming, especially for large-scale data sets. Accordingly, several computation approaches have been developed to effectively and accurately predict lysine ubiquitylation sites. Tung and Ho built a prediction model, UbiPred, by using an informative subset of 531 physicochemical properties and a support vector machine. A new algorithm was then proposed for selecting an informative physicochemical properties subset, which can significantly improve the accuracy [13]. Radivojac *et al*. developed a random forest predictor of ubiquitylation sites, UbPred. In their method, amino acid compositions, physicochemical properties and evolutionary information are first used to represent a protein sequence, and then a t-test attribute selection filter is applied to retain only statistically significant attributes [14]. Cai *et al*. proposed a predictor based on nearest neighbor algorithm. In that algorithm, they extract conservation scores, disorder scores from a protein sequence, and then utilize the maximum relevance and minimum redundancy principle to identify the key features [15]. Nevertheless, the prediction performances of these approaches are not always satisfactory.

In this study, an ensemble computational method is developed to predict lysine ubiquitylation sites based on amino acid sequence features. Firstly, four kinds of useful features, which describe each amino acid of lysine site and its surrounding sites, are extracted from each protein sequence: amino acid composition [16]; evolutionary information [17,18]; amino acid factors [19]; and disorder score [20]. Secondly, in order to reduce the computational complexity and enhance the overall accuracy of the predictor, an effective feature selection method is used to select some optimal feature subsets. Finally, the ensemble classifier is established using the vectors of resulting features subset as input. For the new constructed ubiquitylation sites dataset, the accuracy of the proposed predictor is 76.82% for the training dataset, and 79.16% for the test dataset, which is higher than the state-of-art ubiquitylation site predictor.

Our feature analysis shows that flanking residues will influence the property and structure of a central residue. That is, the environmental information will be helpful to enhance prediction accuracy. In lysine ubiquitylation sites prediction, the position-specific scoring matrix (PSSM) conservation scores play a more important role. The other three descriptors, *i.e.*, amino acid composition, disorder score and amino acid factors, show almost equal relevance to ubiquitylation. When the window size is 21, amino acid residues at location 8, 11, 12, 14, 16 and 20 have much more features in the optimal features subset, compared with the other locations.

## 2. Materials and Methods

### 2.1. Data Sets

In this study, a dataset consisting of 468 ubiquitylation sites from 323 proteins is constructed by retrieving annotated proteins from the UniProt database [21] at [22]. These proteins have been reprocessed in order to avoid homology bias using the program cd-hit [23], so that the sequence identity is lower than 0.6. By mapping the experimentally verified ubiquitylation sites to the corresponding 323 protein sequences, the 920 lysine residues with no annotation of ubiquitylation sites are regarded as non-ubiquitylation sites. The benchmark dataset is then divided into training dataset and test dataset: 65 proteins are randomly selected to construct a test dataset, and the remaining proteins make up the training dataset. According to Tung and Cai’s work [13,15], the best window size for ubiquitylation site prediction is 21, so we adopt it in this study too; with 10 residues located upstream and 10 residues located downstream of lysine residue in the protein sequence. As a result, the training dataset includes 298 ubiquitylation sites and 563 non-ubiquitylation sites, and the test dataset includes 170 ubiquitylation sites and 357 non-ubiquitylation sites.

To evaluate the ensemble classifier’s performance and compare it with existing methods, a publicly available dataset [15] is also adopted here, which includes 14 ubiquitylation sites and 267 non-ubiquitylation sites. In this paper we have called this “independent dataset”. In Table 1, we describe the number of ubiquitylation and non-ubiquitylation sites in each dataset.

### 2.2. Representation of Peptides

In this study, amino acid composition, PSSM conservation scores, disorder scores and amino acid factors are used to transform the peptides into feature vectors.

#### 2.2.1. Amino Acid Compositions

Usually, there are many encoding methods of protein sequence, e.g. amino acid composition [16,24], pseudo amino acid composition method [25] and amino acid identity [13], *etc*. Here we utilize the amino acid composition to represent each peptide, which is based on normalized counts of single or pairs of amino acids. Firstly, each peptide is represented by a feature vector of length 141 that includes 20 features for average amino acid composition and 121 dipeptides. Secondly, in order to reduce the dimensionality of dipeptides, the 20 amino acids are clustered into 11 groups according to similar physicochemical or structural properties [26], and then 121 pairwise combinations are reduced to 66 by classifying the dipeptides with the same amino acid composition into one category.

#### 2.2.2. PSSM Conservation Scores

Evolutionary conservation, one of the most important types of information in assessing functionality in biological analysis, has been used successfully in many studies [17,18]. In biology, conserved sequences are similar or identical sequences that occur within protein sequences, nucleic acid sequences or within different molecules produced by the same organism. Highly conserved proteins are often required for basic cellular function, stability or reproduction. Protein sequences’ evolutionary conservation serves as evidence for structural and functional conservation. So the corresponding position-specific scoring matrix (PSSM) extracted from sequence profiles generated by PSI-BLAST is selected as the second type of feature descriptor in this study. Here, we employ each sample to search and align homogenous sequences from NCBI’s NR database [27] using the PSI-BLAST program [28] with three iterations (−j 3) and e-value threshold for inclusion in multi-pass model 0.0001 (−h 0.0001).

**Figure 1:**
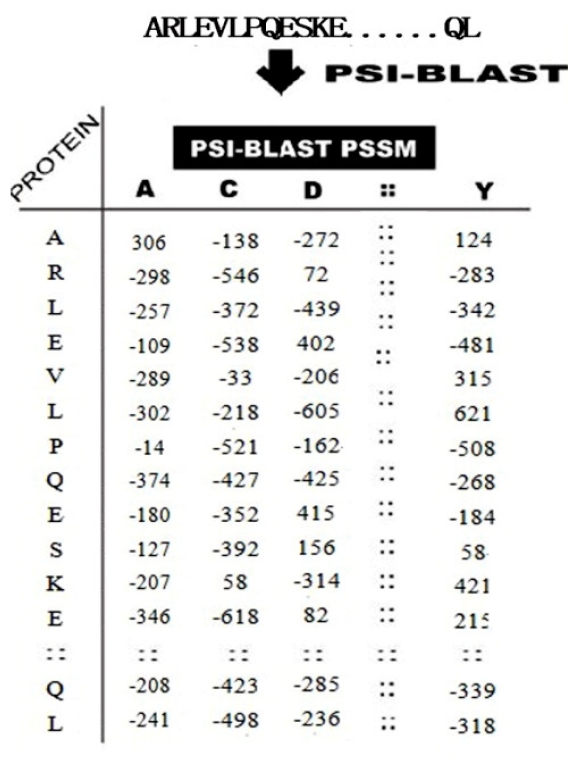
Schematic representation of transformation of each protein sequence into L*20 dimensional position-specific scoring matrix (PSSM); the rows represent the protein residues and the columns represent the 20 amino acids.

It can be seen from Figure 1, the PSSM matrix is composed of L*20 elements, where *L* is the total number of residues in a peptide, the rows of the matrix represent the protein residues and the columns of the matrix represent the 20 amino acids. Each amino acid in the PSSM profiles is encoded by an evolutionary information vector of 20 dimensions using the *i*th row of PSSM. Then we normalize the values of PSSM in range of [0, 1] by using formula (value − minimum)/(maximum − minimum) before we use this PSSM matrix. In order to consider the neighboring effect of residues surrounding each ubiquitylation site, a sliding window of size *w* is utilized to combine the evolutionary information from downstream and upstream neighbors. For an ubiquitylation site K in sequence position *i*, we used a feature vector *P_i_* to represent it. *P_i_* is defined as follows, where *w* is an odd number which stands for the size of sliding window, and *p_[i]_* is the *i*th row of normalized PSSM matrix. The length of vector *P_i_* is w*20.

(1) $$P_{i}=[p_{\left[i-(w-1)/2\right]},\ldots ,p_{\left[i\right]},\ldots ,p_{\left[i+(w-1)/2\right]}]$$

#### 2.2.3. Disorder Scores

In recent decades, the functional importance of disorder regions has been increasingly recognized [29,30]. Protein disorder in the nonglobular segments allows for more modification sites and interaction partners, and is of great importance to predict protein structures and functions [29,31,32]. In this paper, we use the disorder score calculated by VSL2 [33] to represent each amino acid disorder status in the given protein sequence. The VSL2 predictor can accurately identify both long and short disordered regions [34,35]. The disorder score features are composed of the disorder scores of the lysine site and its surrounding sites.

#### 2.2.4. Amino Acid Factors

The structure and function of proteins are largely dependent on the composition of various properties of each of the 20 amino acids. The individual amino acid physicochemical properties have been successfully used in lysine ubiquitylation identification [36,37]. AAIndex [38] is a well known database of amino acids’ biochemical and physicochemical properties. Atchley *et al*. [19] have conducted multivariate statistical analysis on this database. They summarized this and provided five highly interpretable and multi-dimensional numeric indices that represent electrostatic charge, codon diversity, molecular volume, secondary structure, and polarity. Thus, we use these five numerical index scores (also called “amino acid factors”) to encode each amino acid in this study.

#### 2.2.5. Feature Space

For every sample in the dataset, its feature space is composed of the features of AA compositions, PSSM scores, amino acid factors and disorder scores. Totally, there are 627 features to be encoded in a sample, including 86 amino acid composition features, 420 (20 × 21 = 420) PSSM conservation score features, 100 (20 × 5 = 100) amino acid factors features and 21 disorder score features.

### 2.3. Feature Selection Based on Normalized Conditional Mutual Information

Usually, the most popular feature selection methods find a single features subset whose discriminative capability is limited for classification purpose [39]. In fact, there are many feature subsets with good discriminative power, so we use an effective feature selection method [40], Feature Selection based on Normalized Conditional Mutual Information (FSNCMI), to predict lysine ubiquitylation sites by manipulating multiple feature subsets simultaneously. Unlike other ensemble methods which use different classifiers or different sample subsets to strengthen the final prediction accuracies, FSNCMI obtains multiple feature subsets by the same selection technique with different starting points in its research process.

To measure the information shared by two features, mutual information (MI) is used here, defined as follows:

(2) $$I(X;Y)=\sum_{x\in dom(X)}\sum_{y\in dom(Y)}p(x,y)\;\text{log}\frac{p(x,y)}{p(x)p(y)}$$

where *X* and *dom*(*X*) are discrete random variable and its domain, *p*(*x, y*) is the joint probabilistic density, and *p*(*x*) and *p*(*y*) are the marginal probabilistic densities. According to the concept of Shannon entropy, joint entropy and conditional entropy in information theory [41], the following equations stand:

(3) I(X;Y)=H(X)-H(X/Y)=H(Y)-H(Y/X)=H(X)+H(Y)-H(X,Y)

Similarly, the conditional mutual information between *X* and *Y* given *Z* is defined as:

(4) I(X;Y/Z)=H(Y/Z)-H(Y/X,Z)

where *Z* is a discrete random variable.

Equation 4 represents the reduction of uncertainty of *Y* with respect to *X*, when *Z* is known. From the definition of *conditional mutual information*, we know that *I*(*f;C*/*FS*) can be used to measure the information amount shared by the feature *f* and the class labels C. Yet this information has not been captured by the already selected features *FS*. Therefore, the conditional mutual information *I*(*f;C*/*FS*) can be taken as the evaluation criterion *J*(*f*) of feature selection to evaluate the significant degree of feature *f*, and then at each step, the feature with maximal *I*(*f;C*/*FS*) will be selected. Normally, *FS* is replaced by one of its member *f_s_* to deal with the problem that the estimation of *I*(*f;C*/*FS*) by multivariable dense distribution is usually unfaithful [42]. Thus we have

(5) $$J(f)=\underset{f_{s}\in FS}{\text{arg}\;\text{min}}\;I(f;C/f_{s})$$

Note that the criterion of conditional mutual information may tend to choose the feature with more concrete values [43], so we normalize it by *H*(*f*,*C*) and refer to it as normalized conditional mutual information, *i.e.*,

(6) $$J^{\prime }(f)=\underset{f_{s}\in FS}{\text{arg}\;\text{min}}\frac{I(f;C/f_{s})}{H(f,C)}$$

Based on the above analysis, the normalized form of conditional mutual information can also be used to evaluate the correlation between features and target classes when other features are known. On the ground of this criterion, the ensemble feature selection method can be induced by using normalized conditional mutual information, which is described as follows:

**Algorithm 1.** FSNCMI: feature selection based on normalized conditional mutual information.

**Input:** An ubiquitylation sites dataset M = (*D*, *F*); the index of starting point *t*; the number of selection features *k*;

**Output:** A set of selected features *FS_i_*;

**Table:** 

(1)	Initialize related parameters, *FS_i_* =Ø, F = *F*, *t* = 0;
(2)	**For** each feature *f* ε F **do**
(3)	calculate its mutual information with the target classes *C*;
(4)	Sort them in a descending order;
(5)	*FS_i_* = {*f_t_*}, F = F − {*f_t_*}, where *f_t_* is the starting point;
(6)	**While** |*FS_i_*| < *k***do**
(7)	**For** each feature *f* ε F **do**
(8)	Calculate its criterion *J*(*f*) according to Equation.(4);
(9)	If *J*(*f*) = 0 then F = *F* − {*f*};
(10)	Select the gene with the largest *J*(*f*);
(11)	*FS_i_* = *FS_i_* ∪{*f*}, F = *F* − {*f*};
(12)	**End**

This algorithm works in a straightforward way. For the stopping condition k, we choose the strategy that when the difference of *J*(*f_i_*) with *J*(*f_i-1_*) is lower than a very small value, the iterative procedure is terminated. For the starting point, we choose *t* = 0, since the top-ranked features have higher mutual information and they may highly correlate with each other. To some extent, this will strengthen the stability and classification performance of the classifier [42].

### 2.4. Random Forests Classification

Random Forests (RF) is a classification algorithm combining ensemble tree-structured classifiers [44], which has been successfully used to deal with some problems in the bioinformatics area [45–47]. In RF, each tree is grown using a subset of the possible attributes in the input vectors [48]. The results from [46] showed that combining multiple trees produced in randomly selected subspaces can enhance the prediction performance. The RF is useful for estimating prediction errors. The prediction error is estimated by using an out-of-bag (OOB) sample. For each RF tree, the OOB sample including approximately one-third of the training dataset is applied to test the decision tree constructed by using the remaining training dataset with no pruning procedure. Finally, the overall prediction error is then calculated by combining results from the trees via voting, which can avoid over fitting on the training set while preserving maximum accuracy. The RF algorithm is available via the link at [49]. Recently, the RF code for the MATLAB windows is also available at [50], which has two functions, one is “classRF-train” for establishing a prediction model, and the other is “classRF_predict” for predicting the test dataset using the prediction model. The classifier in this study is developed based on this RF.

### 2.5. Model Building

**Figure 2:**
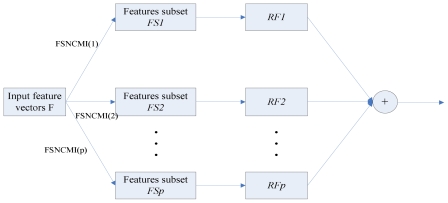
The framework of the ensemble model.

The ensemble classifier can combine several decisions induced by the individual classier into one in some way. Compared with traditional methods, ensemble classifier can effectively improve classification performance, reliability and stability of individual classier. Figure 2 describes the whole framework of our model.

Figure 2 shows the main framework of our method. Firstly, each peptide is transformed to vectors by using amino acid composition, PSSM conservation scores, disorder scores and amino acid factors features. Secondly, FSNCMI feature selection method is utilized to extract *P* informative feature subsets. After that, these corresponding RF predictors will be aggregated into a consensus using the majority voting strategy.

### 2.6. Evaluation

Jackknife test [51] is a rigorous and objective statistical test, and has been widely used to examine the performance of various predictors [52–55]. Therefore we use it to evaluate our method as well, where proteins are singled out from the dataset one by one as a testing protein and the classifier is trained by the remaining proteins. Besides the jackknife test on training set, we also utilize sub-sampling (e.g., 5- or 10-fold cross validation) and an independent test [56] to evaluate our model. Since the number of ubiquitylation sites and non-ubiquitylation sites are imbalanced in both the training set and the independent set, the Matthews’s correlation coefficient (*MCC*) is used here to objectively measure the performance of our ensemble classifier. MCC is usually regarded as a balanced measure to process imbalanced data [53]. Meanwhile, sensitivity (*S_n_*), specificity (*S_p_*) and accuracy (*AC*) are also used. These parameters are defined by the following formulas:

(7) $$\begin{array}{l} S_{n}=\frac{TP}{TP+FN}\\ S_{p}=\frac{TN}{TN+FP} \end{array}$$

where *TP*, *TN*, *FP* and *FN* stand for true positive, true negative, false positive and false negative, respectively.

## 3. Results and Discussion

### 3.1. Prediction Performance of Our Method

Here, we evaluate the prediction performance of the ensemble classifier on the training set constructed in this study which consists of 298 ubiquitylation sites and 563 non-ubiquitylation sites. In our model, the number of features (stopping condition k in Algorithm 1) is an important parameter in the implementation of the ensemble predictor, so we should assign *k* with an appropriate value. We compare the difference of *J*(*f_i_*) with *J*(*f_i-1_*), and find that when *i* = 13 (*i* = 1 to 627), the difference is lower than the threshold 0.015. So the number of selected features in the ensemble selector is set to be 12. Such a selection is reasonable, because f_13_ brings little information to any already selected features in FS.

**Figure 3:**
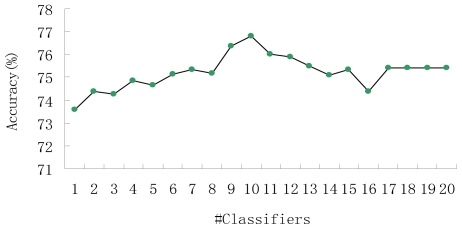
The relationship between the prediction performance and the quantity of base classifiers.

Moreover, for prediction performance, the quantity of base classifiers (Qbc) is another aspect associated with ensemble classifier. Therefore, in the implementation of ensemble model, Qbc must also be considered. Intuitively, the prediction capability of ensemble model is highly affected by the number of base classifiers and, the more base classifiers contained in an ensemble selector, the higher the accuracy obtained by the model. To illustrate this kind of relationship between Qbc and prediction performance, we run our model with various numbers of Qbc. The results on the training set constructed in this study which consisted of 298 ubiquitylation sites and 563 non-ubiquitylation sites, are presented as Figure 3. As can be seen in the figure, the prediction performance of ensemble model becomes stable when Qbc reaches the point where Qbc equals 16. Although the increasing of Qbc will improve the classification performance and the stability of the ensemble model to some extent, it is not appropriate to employ as many base classifiers as possible. The reason for this is that when Qbc reaches a certain value, the performance will only increase a little, but the computational cost of building base classifiers will increase abruptly. As is shown in Figure 3, the ensemble model obtains the highest accuracy of 76.82% when Qbc is 10.

Since the evaluation criterion of FSNCMI is normalized conditional mutual information, the maximum relevance and minimum redundancy principle (mRMR) [57] feature selection method, which is based on mutual information, is also taken as the base line. mRMR chooses those features which has more relevance to the class labels and less redundancy to the selected features at the same time. In our experiments, mRMR chooses the same features as FSNCMI, for both the training dataset and the independent dataset. All experiments are performed and report the *S_n_*, *S_p_*, *AC* and *MCC*. The comparison results of the two feature selection methods by jackknife test on the training set are shown in Table 2. The comparison results of the two feature selection methods by 5-fold cross validation test on the test dataset are shown in Table 3. For the training set and the independent test set, our ensemble predictor achieves accuracies of 76.82% and 79.16%; higher than the results obtained by using the mRMR feature selection method by 9.4% and 9.96% respectively. This may be due to the use of an effective feature selection method (FSNCMI), which can manipulate multiple feature subsets simultaneously.

Recently, two groups managed to identify a large number of endogenous ubiquitylation sites in human cells using mass spectrometry [58,59]. We picked out 300 lysine ubiquitylation sites downloaded from [58], and each ubiquitylation site in this dataset does not appear in the training dataset or the test dataset. Similarly, the 300 ubiquitylation residues with no annotation of ubiquitylation sites are regarded as non-ubiquitylation sites. The ensemble model obtains an accuracy of 75.26% and MCC of 0.623. In the future, we will use these identified sites as the training dataset to further improve the prediction accuracy of our ensemble model.

### 3.2. Comparison with Existing Methods

In this section, the proposed ensemble predictor is further compared with three recently reported predictors [13–15] on a publicly available dataset [15], which includes 14 ubiquitylation sites and 267 non-ubiquitylation sites. The number of ubiquitylation sites and non-ubiquitylation sites in this dataset are highly imbalanced, and this situation is close to reality. The compared results are shown in Table 4. As can be seen from the table, the ensemble predictor proposed in this study obtains an *MCC* of 0.153, higher than the other three methods with an accuracy of 71.32%. In Table 4, “NA” means that the corresponding terms are until now unknown.

### 3.3. Feature Analysis

**Figure 4:**
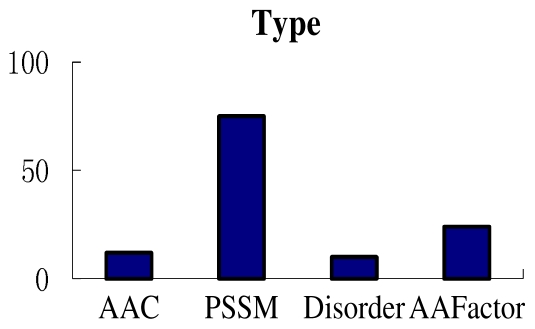
The number of each type of feature in the 10 selected subsets.

As is described in Section 2.2, we utilize four kinds of attributes to represent a peptide: amino acid compositions; PSSM conservation scores; disorder scores; and amino acid factors. In this section, feature analyses are performed on the training dataset. Firstly, the number of each type of features in the selected 10 subsets is counted and shown in Figure 4. There are 12 amino acid compositions features, 75 PSSM conservation scores features, 10 disorder scores features and 24 amino acid factors features in the selected subsets. From Figure 4, we can conclude that the PSSM conservation scores play the most important role in the lysine ubiquitylation prediction. In addition, it should be noted that most disorder scores features are extracted from site 13, this means that the disorder status of amino acid around the ubiquitylation site may affect the ubiquitylation process.

**Figure 5:**
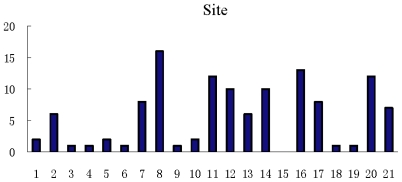
The number of all features on each site in the 10 selected subsets.

Secondly, the number of features in each amino acid site taken from the 10 selected subsets is counted and shown in Figure 5. It is obvious that there are much more features in the selected subsets on sites 7, 8, 11, 12, 14, 16, 20 and 21 than on the other sites. This phenomenon may explain why Tung and Ho [13] found the best window size for ubiquitylation sites prediction to be 21.

**Figure 6:**
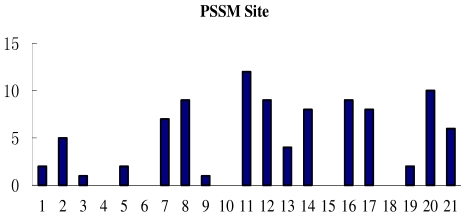
The number of PSSM features on each site in the 10 selected subsets.

Finally, the number of PSSM features in each amino acid site taken from the selected 10 subsets is counted and shown in Figure 6. It can be seen that the conservation of lysine residue plays a key role in the ubiquitylation process; moreover, the nearby sites 8, 12, 14 and remote sites 2, 7, 16, 17, 20 have more PSSM conservation scores features than the others.

## 4. Conclusions

Prediction of lysine ubiquitylation sites is important to understand the molecular mechanism of lysine ubiquitylation in biological systems. Though some researchers have focused on this problem, the accuracy of prediction has still not been satisfied. In this study, we develop an ensemble predictor for the prediction of lysine ubiquitylation sites based on a new feature selection method using the information of sequence conservation, amino acid composition, amino acid physicochemical properties and residue disorder status. The accuracy of our predictor is higher than those of state-of-art ubiquitylation sites prediction tools. Experimental results have shown that our method is very promising and may be a useful supplement tool to existing methods. Moreover, the conclusions derived from this paper might help to understand more about the ubiquitylation mechanism and guide related experimental validations.

Since user-friendly and publicly accessible web-servers represent the future direction for developing more practically useful models, simulated methods or predictors, in our future work we will attempt to provide a web-server for the method presented in this paper.

## Figures and Tables

**Table 1:** The number of ubiquitylation and non-ubiquitylation sites in each dataset.

Dataset	No of ubiquitylation sites	No of non-ubiquitylation sites
Training dataset	298	563
Test dataset	170	357
Independent dataset	14	267

**Table 2:** The performance comparison of two feature selection methods on the training dataset.

Method	*S_n_* (%)	*S_p_* (%)	*AC* (%)	*MCC*
mRMR [57]	64.76 ± 2.12	68.21 ± 3.52	67.42 ± 1.37	0.282 ± 0.13
This paper	76.85 ± 1.84	76.91 ± 2.09	76.82 ± 1.03	0.519 ± 0.08

**Table 3:** The performance comparison of the two feature selection methods on the test dataset.

Method	*S_n_* (%)	*S_p_* (%)	*AC* (%)	*MCC*
mRMR [57]	51.68 ± 1.35	74.22 ± 0.92	69.20 ± 1.06	0.229 ± 0.09
This paper	72.61 ± 2.34	81.27 ± 0.76	79.16 ± 0.98	0.503 ± 0.07

**Table 4:** The performance comparison of different predictors on the independent dataset.

Predictor	*S_n_* (%)	*S_p_* (%)	*AC* (%)	*MCC*
mRMRPred [15]	34.34	79.67	68.34	0.139
UbiPred [13]	NA	NA	NA	0.135
UbPred [14]	NA	NA	NA	0.117
This paper	57.14 ± 1.39	74.15 ± 0.95	71.32 ± 1.26	0.153 ± 0.06
